# Dry Eye Disease: What Is the Role of Vitamin D?

**DOI:** 10.3390/ijms24021458

**Published:** 2023-01-11

**Authors:** Maurizio Rolando, Stefano Barabino

**Affiliations:** 1 Ocular Surface and Dry Eye Center, Is.Pre Oftalmica, 16129 Genoa, Italy; 2 Ocular Surface and Dry Eye Center, ASST Fatebenefratelli-Sacco, Ospedale Sacco-Università di Milano, 20157 Milan, Italy

**Keywords:** vitamin D, dry eye disease, dry eye syndrome, dry eye therapy, systemic supplementation

## Abstract

Dry eye disease (DED) is a multifactorial condition resulting from reduced tear secretion from the lacrimal glands, increased tear water evaporation or the production of poor-quality tears. Such tear instability can lead to inflammation and damage of the ocular surface, as well as to abnormal nociception. Historically, tear substitutes and corticosteroids have been the bastion of DED therapy, but a substantial number of patients still suffer from residual symptoms even after being treated with traditional treatments. Aiming to find safe and effective alternative therapies, recent efforts have been focused on the role of vitamin D in the cellular physiology of the eye. Possibly because of its positive effect in modulating the immune and inflammatory responses, the systemic supplementation of vitamin D seems, indeed, to be an effective therapeutic strategy, especially, but not only, for patients affected by DED that does not respond to conventional treatments. In this context, this review focuses on the literature reporting on the pathogenesis and treatment of DED, with a special emphasis on the recent investigations reporting on the potential role of the systemic administration of vitamin D as a therapeutic approach in the management of such condition.

## 1. Introduction

The term vitamin D indicates a group of fat-soluble secosteroids that are naturally present in few foods and can also be produced endogenously in the skin. In humans, vitamin D3 (also called cholecalciferol) and vitamin D2 (ergocalciferol) represent the two most important compounds of this group [1]. Vitamin D is not strictly a vitamin, since, given its molecular steroid-like structure, as well as its wide range of endocrine actions, it may be considered a hormone. Its main function is to promote calcium absorption in the gut and maintain suitable calcium and phosphate levels in the serum to support normal bone mineralization and health [2,3,4,5].

Vitamin D endogenously synthesized upon sun exposure or obtained from foods and supplements is biologically inert and must undergo two enzymatic hydroxylation steps to be activated. The first hydroxylation, which converts vitamin D to 25-hydroxyvitamin D (25(OH)D) (also called calcidiol), occurs in the liver. Differently, the second hydroxylation takes place mainly in the kidneys and produces the physiologically active 1,25-dihydroxyvitamin D (1,25(OH)2D) (also called calcitriol) [1,5]. Considering its quite long circulating half-life (i.e., 15 days), the serum concentration of 25(OH)D is currently the main indicator of the vitamin D status, as it reflects the amount of vitamin D produced endogenously, as well as that derived from foods and supplements [6].

Serum concentrations determining a 25(OH)D deficiency have not been conclusively identified. According to an expert committee of the Food and Nutrition Board (FNB) at the National Academies of Sciences, Engineering, and Medicine (NASEM), people with serum 25(OH)D levels of less than 30 nmol/L should be considered at risk of vitamin D deficiency, while people in the range from 30 to 50 nmol/L should be warned of being potentially at risk of inadequacy. In general, concentrations of 50 nmol/L or more are considered sufficient for the majority of people [5,6,7,8,9]. Since only a relatively small number of foods (e.g., egg yolks, red meat, liver, and oily fish), which are generally not part of most people’s daily diet, contain vitamin D, the contribution from food sources is quite limited. For this reason, it is often necessary to prescribe vitamin D supplements to people who are experiencing vitamin D deficiency [6]. 

Based solely on the quantity of vitamin D required to regulate the homeostasis of calcium and phosphate, the Institute of Medicine recommends for healthy people a vitamin D intake of 4000 IU per day [5,6]. Nevertheless, such a level of intake might not be sufficient if one considers the putative non-calcemic functions of vitamin D or, even more critically, if disease states requiring vitamin D therapy (e.g., osteoporosis, diabetes mellitus, and obesity) are taken into account [10,11]. In these pathological cases, the guidelines of the Endocrine Society suggest an intake level of 10,000 IU per day and even greater levels if specific cases of malabsorption syndrome come into play [6]. 

The reason why such different levels of intake need to be recommended is grounded in the fact that nutrients, as well as drugs, follow a sigmoidal dose–response curve. This characteristic shape has two major implications that are relevant for the purpose of prescribing a proper dose of vitamin D: (a) response to a change in intake strictly depends on the individual’s starting status (drug or nutrient baseline level), and a (b) change in intake should be large enough to cover almost all the response area [12]. Concerning point (a), physicians providing vitamin D to patients should calculate the adequate amount of vitamin D on the basis of the patient’s baseline level and keeping in mind the nonlinearity of the dose–response curve. Indeed, within physiological ranges, the same dose of vitamin D might provide no response or a significant response, depending on whether the patient’s baseline level lies on the minimum/maximum plateau regions of the sigmoid or on the mid-region of the curve (almost linear) [13]. Furthermore, point (b) suggests that, especially in cases of severe deficiency or when distant body districts are implicated (e.g., eyes), resorting to pharmacological doses of vitamin D (>10,000 IU) might constitute the only therapeutical way to achieve a beneficial response. 

Various health problems might require the prescription of vitamin D as a way to contrast its deficiency. In recent years, vitamin D deficiency has been linked not only to its well-recognized role in musculoskeletal disorders (e.g., rickets or osteomalacia) and mental health [14] but also to other conditions, including malignancies [15,16,17], type 2 diabetes mellitus and metabolic syndrome [18], hypertension and cardiovascular diseases [19,20], and immune and autoimmune disorders [21], as well as eye disorders [22,23].

Focusing on the role of vitamin D in the eye, immunohistochemical experiments have found that, in the human eye, the vitamin D receptor (VDR) is expressed in the retinal pigment epithelium, ciliary body, lens, and epithelium of the cornea, as well as in the retinal photoreceptors, ganglion cell layer, and corneal endothelium [24]. Vitamin D hydroxylases (CYP27B1, CYP27A1, CYP2R1, and CYP24A1) were later shown to be present in endothelial, corneal epithelial, nonpigmented ciliary body epithelial, scleral fibroblasts, and adult retinal pigment epithelial cell lines, which could also convert 25D into the functionally active 1,25D [25,26]. Such results suggest that several ocular cell types are endowed with the ability to metabolize and activate vitamin D. Nevertheless, as reported in a recent review, clinical studies supporting the therapeutic effectiveness of vitamin D topical formulations in treating ocular diseases are not present in the literature [27]. Additional sources of vitamin D are likely to be the aqueous humor, the vitreous humor, and tear film. Indeed, in these districts of the rabbit eye, vitamin D metabolites were found and their level increased after the oral supplementation of vitamin D, further emphasizing that the eye may represent a site of extrarenal vitamin D production [25,28]. 

These observations and an increasing body of evidence strongly suggest that vitamin D plays a role in the eyes’ cellular physiology and that it is likely important for maintaining ocular health. For the sake of clarity, in this review, we will focus on the pathogenesis and treatment of dry eye disease, emphasizing the potential role of the systemic administration of vitamin D as a therapeutic approach in the management of such condition.

## 2. Dry Eye Disease

Dry eye disease (DED) is a multifactorial condition that comes into play when tears provide inadequate lubrication for the eyes or their content becomes toxic for the eye surface. Dry eye disease results from reduced tear secretion from the lacrimal glands, increased tear water evaporation, or the production of poor-quality tears [29,30,31]. This tear instability leads to inflammation and damage of the ocular surface and abnormal nociception, resulting in ocular discomfort and impaired visual performance [29,32,33,34,35]. The most common symptoms of DED include persistent itchiness, redness, dryness, and a burning sensation, associated with the continued sensation of a foreign body in the eye, sensitivity to light, and blurred vision [36]. 

Dry eye disease is one of the most frequent ocular disorders worldwide [37,38] and a significant public health issue [39,40]. The burden of DED on patients is considerable. It affects not only visual functions but also daily life activities, both from a social and physical point of view, productivity in the workplace, and, more generally, it affects the patient’s quality of life [41,42]. In epidemiological studies, the prevalence of DED in people over 40 years of age ranges from 5 to 50 percent, and the disorder seems to be associated with age and female gender. Indeed, older women are more likely to suffer from symptoms associated with dry eye [43]. The large variation observed in DED prevalence is presumably due to discrepancies in definitions, diagnostic criteria, and study populations that are typically reported in the literature. 

The association between older age and DED is supported by a study showing that 30% of elder care facilities residents suffer from the disease and that medications and frailty status may enhance the risk of suffering from dry eyes [44]. In addition to older age and female gender, a number of risk factors, such as medications, medical conditions, and environmental agents, can lead to DED onset. Important environmental factors are exposure to smoke, windy and dry climates that increment tear evaporation, and air pollution [43]. Activities leading to dysregulated blinking such as staring at screens for long periods, together with the prolonged use of contact lenses and refractive eye surgery, can also contribute to DED. Reduced levels of tear production are also associated with the intake of several drugs, including blood pressure medications, decongestants, antihistamines, and antidepressants. Local conditions such as eyelid deformity, meibomian gland dysfunction, blepharitis, and conjunctivochalasis are recognized to increase the risk of DED, and systemic disorders such as diabetes mellitus, chronic pain syndrome, rheumatoid arthritis, anxiety, depression, thyroid disease, irritable bowel syndrome, allergic diseases, and hyperlipidemia have been associated with DED [45,46,47,48,49,50]. 

Interestingly, many of these local and systemic conditions have been linked to low serum levels of 25(OH)D or inadequate sunlight exposure [51,52,53,54,55]. Recently, evidence for the potential role of vitamin D in influencing the onset and progression of DED has been accumulating, based both on preclinical data and on clinical reports suggesting that a systemic supplementation of vitamin D may be beneficial.

After a propaedeutic explanation of the pathogenic mechanisms underlying DED, the possible role of the administration of vitamin D as a therapeutic approach in the management of DED will be further justified and discussed in the following sections, providing an in-depth review of the literature reporting on the association between vitamin D deficiency and several aspects of the disease.

## 3. Pathogenic Mechanisms of DED

In the last 15 years, an increasing amount of research has clearly indicated that inflammation plays a pivotal role in the etiopathogenesis of DED [56,57]. Studies in vitro and in animal models, as well as in humans, strongly support the role of inflammation in the pathogenesis of DED [57]. Although DED may be triggered by inadequate eye lubrification resulting from a variety of factors (as reported in the previous section), the disease is then sustained by a concatenation of events involving tear film instability, ocular surface inflammation, and hyperosmolarity as the main actors [58,59,60,61]. 

The ocular surface immune response is, indeed, a highly sophisticated process, which is fundamental to protect the ocular surface. Therefore, circumstances leading to the failure of such a regulated mechanism can lead to DED [59,62,63,64]. Accordingly, numerous inflammatory cytokines, matrix metalloproteinases (MMPs), and chemokines display elevated levels in the tear film of patients with DED. For these reasons, DED-related inflammatory biomarkers are considered clinically valid to confirm the diagnosis of DED, as well as to grade its severity [56,65,66,67]. Along with the quantification of DED-related inflammatory biomarkers, tear osmolarity—indirectly quantified by means of tear impedance measurements—has been regarded as one of the best global markers of DED [68,69]. In this regard, a value of 305 mOsm/L has been suggested to be an appropriate threshold to distinguish normal eyes from the early stages of DED [70]. Differently, more clearly diagnosed and advanced stages of DED seem to be characterized by values of tear osmolarity greater than 316 mOsm/L [71].

Going deeper into the immune processes occurring at the ocular surface, an innate response can be triggered by specific stress to the surface of the cornea, and the resulting activation of mitogen-activated protein kinases can then stimulate the transcription of nuclear factor kappa B (NF-κB), chemokines, and MMPs [72,73]. As a part of the innate response process reacting to the insult, the immune cells at the ocular surface also become activated. Tumor necrosis factor alpha and interleukins 1 and 6 stimulate the maturation of antigen-presenting cells (APCs), which, after migrating to regional lymph nodes, promote the transition to an adaptive immune response [74]. Indeed, APCs are involved in the differentiation of various types of mature T cells—specifically produced against the relevant antigens—which, in turn, travel to the sites of inflammation, the conjunctiva, and the surface of the eyes [75,76,77]. Once activated, T cells secrete proinflammatory factors that sustain the immune response [58,63]. The interruption of such inflammatory processes is normally managed by immunoregulatory mechanisms, such as the secretion of cortisol by epithelial cells of the ocular surface [78], goblet cell secretion of transforming growth factor beta, and programmed death-ligand 1 regulation of activated effector T cells [77,79]. When such immunoregulatory controls are suppressed, the conditions to initiate a vicious cycle are set. Indeed, the immune response is amplified, resulting in the enhanced activity of mature APC and boosted production of T cells. As a consequence, proinflammatory cytokines are released by T cells, de facto worsening the inflammation and damage status and thus reinitiating the cycle of the innate immune response [58,80,81].

As the other key player in the pathogenesis of DED, tear film is composed of water, electrolytes, mucins, and a number of proteins and lipids. It consists of lipids covering a hydrated mucus layer that extends across the epithelial glycocalyx [82]. The instability of tear film is commonly caused by desiccation of the corneal surface. When the epithelial cells of the cornea are damaged, the anchorage of mucins to the ocular surface is impaired, resulting in tear film alteration. In the absence of mucins, the corneal surface becomes hydrophobic, thus repelling aqueous components of the tear film. These events further destabilize the tear film and increase tear evaporation and osmolarity. In such a scenario, a condition of chronic epithelial stress is established, and the typical inflammatory cascade of DED is initiated [74].

## 4. Treatment of DED

Since the loss of tear film homeostasis is a crucial event in the pathogenesis of DED, as it triggers the vicious circle between hyperosmolarity of the tear film and inflammation at the ocular surface that underlies the onset of the disease [80], the aim of DED treatment is to restore this homeostatic process by breaking this vicious cycle. This is achieved in two different ways, either by preventing the corneal surface from drying out or by suppressing the inflammatory response of the eye.

As a strategy to prevent corneal surface desiccation, historically, tear substitutes or artificial tears have been the bastion of DED therapy. They are available in various topical formulations, including lubricants, drops, ointments, and gels. Tear substitutes are designed to supplement the inadequate production of tears and to provide the necessary ocular lubrication, eventually reducing tear evaporation and stabilizing the tear film, which, in turn, prevents desiccation of the eye surface [83]. Polymers such as hyaluronic acid—a highly hydrophilic, non-sulfated, disaccharide glycosaminoglycan occurring naturally in the human body—are commonly used to increase the stability of the tear film. Due to its capacity of increasing viscosity, improving retention time, and optimizing hydration and lubrication of the ocular surface, hyaluronic acid can be regarded as an essential component of tear substitutes [84,85]. 

To control and reduce ocular surface inflammation, topical corticosteroids are typically used as potent inhibitors of several inflammatory mediators. Indeed, they effectively interrupt the vicious cycle of sustained inflammation [75] by suppressing MMPs, acute-phase cytokines IL-1 and TNF-α, chemokines, and ICAM-1 [86], as well as reducing leukocyte infiltration in inflamed ocular tissue [87,88]. Due to their action on specific aspects of inflammation, other molecules that are used to contrast inflammation, alone or combined with topical corticosteroids, are o-mega-3 fatty acids, topical cyclosporine A, tacrolimus, and topical lifitegrast [89,90,91,92,93,94]. 

Unfortunately, the aforementioned approaches may not be resolutive for DED treatment, and a substantial number of patients, after being treated with the current medical therapies, still suffer from residual symptoms [95,96]. The main limitations of tear substitutes are consistent with their palliative nature. Indeed, as expected from their composition and intended purpose, they do provide eye lubrification and, thus, immediate relief, but they do not represent a solution capable of treating the inflammation underlying DED [97]. Concerning topical corticosteroids, their limitations in treating DED are reverted with respect to artificial tears. Indeed, if, from the one side, they represent an extremely efficient strategy to suppress DED-related inflammation, from the other side, their long-term use has been associated with several adverse events, including cataracts and ocular hypertension [97,98].

Therefore, when such treatments fail to provide a safe and complete restoration of the physiological ocular status, alternative treatment options might represent a successful strategy. In this regard, recent efforts to identify alternative approaches for preventing and treating DED have been focused on vitamin D [25,99]. This is mainly due to its involvement in regulating multiple processes of the immune inflammatory response. For instance, 1,25(OH)2D—the active form of vitamin D—is capable of inhibiting cell proliferation and stimulating differentiation [100], as well as of modulating inflammatory cytokines that are dependent on NF-κB activity in numerous cell types [101]. Most importantly, considerable scientific evidence shows that 1,25(OH)2D is a potent immune system and inflammation modulator that enhances the innate immunity and inhibits the development of autoimmunity [102,103,104]. Conversely, the integrity of the immune system might be compromised by a deficiency status of vitamin D, leading to inadequate immune responses. Due to these remarkable effects on the immune system, it is likely that vitamin D might also influence the development of DED, as this is linked to dysfunctional immune regulation and inflammation. In such a scenario, patients suffering from DED might benefit from a systemic administration of vitamin D, which, therefore, could be used as an adjuvant therapy for this disorder.

## 5. Evidence Linking DED and Vitamin D

Several ocular pathologies—which encompass uveitis, retinoblastoma, diabetic retinopathy, age-related macular degeneration, myopia, and dry eye—have been associated with vitamin D deficiency and variations of the genes regulating its metabolism [105].

Initial hints of the crucial role of vitamin D in the eye came from preclinical research. These studies revealed the presence of VDR (vit D Receptor) and enzymes required for vitamin D metabolism in many cells of the eye, suggesting that vitamin D acts as a paracrine/autocrine regulator [25,26]. This hypothesis is supported by experimental evidence showing that retinal and corneal cells can convert vitamin D to its active form and are able, after UV irradiation, to synthesize vitamin D from exogenous 7-dehydrocholesterol. Moreover, vitamin D metabolites were found in tear fluid and in the aqueous and vitreous humors [26,28], implying an eye-specific production of vitamin D. Consistently, the 25(OH)D levels in human tears were reported to be higher than the corresponding serum concentrations [106]. Moreover, experiments on mice showed that the healing of the corneal epithelium was impaired after VDR gene inactivation and that vitamin D had a beneficial effect on the barrier function of the corneal epithelium [25,107,108], thus indicating a critical role of vitamin D in maintaining the corneal integrity.

The function of vitamin D as a potent modulator of the innate and adaptive immune system in the eye has been supported by in vivo studies showing that vitamin D is able to inhibit corneal inflammation by suppressing the migration of Langerhans cells into the cornea [109], curbing the excessive production of proinflammatory mediators such as interleukins and TNFα [110,111] and attenuating the inflammation in a dry eye model in rats [112]. The anti-inflammatory properties of vitamin D were also observed in a study focusing on the mouse retina. In this investigation, the number of activated macrophages was significantly reduced by subcutaneous injections of calcitriol, which eventually attenuated chronic inflammation [113].

Recently, the role of vitamin D in DED pathogenesis has been investigated in clinical studies addressing the association between vitamin D deficiency and several aspects of the disease. A PubMed search (carried out in 2021) using the terms “vitamin D deficiency”, “dry eye disease”, and “dry eye syndrome”, restricted by selecting the filters “clinical trial” and “Meta-Analysis”, yielded a relatively small number of studies (Table 1), which are described below.

A deficiency of vitamin D was linked to a decreased tear break-up time, lower Schirmer test values, tear hyperosmolarity, and tear film dysfunction, eventually suggesting a probable association with dry eye symptoms [114,115]. Similar results were also reported in a population of premenopausal women with vitamin D deficiency who resulted in also being affected by impaired tear stability, suggesting that vitamin D exerts a protective role against the development of DED [116]. Furthermore, there is evidence that vitamin D serum levels correlate not only with tear stability but also with tear secretion [117]. Reduced vitamin D serum levels have also been linked to exaggerated symptoms in patients with mild dry eye signs. Interestingly, these patients showed an altered cytokine profile in the tears, with increased levels of proinflammatory molecules [33]. Genetic evidence has also been collected linking vitamin D with DED. In a study including 64 DED cases and 51 controls, DED occurrence resulted in being associated with single-nucleotide polymorphisms in the VDR genes [127].

A significant association between low serum concentrations of vitamin D and the occurrence of DED was established in a case–control study involving 70 patients with DED and 70 matched controls [118] and in a large Korean adult population study based on data from the fifth Korean National Health and Nutrition Examination Survey (KNHANES). In the latter study, DED was also associated with inadequate sunlight exposure [119]. Taken together, such results indicate that an adequate sunlight exposure or vitamin D supplementation may be beneficial for patients suffering from DED. In contrast to these findings, a cross-sectional study of the Korean population using data from the KNHANES, as well as another Korean study that evaluated data from the Study Group for Environmental Eye Disease (SEED), revealed no significant association between vitamin D serum concentrations and DED [120,121]. However, the results of a recent meta-analysis examining most of the available data on the association between vitamin D and dry eye showed that the serum vitamin D levels were lower in patients suffering from DED than in healthy subjects and that vitamin D deficiency was associated with increased dry eye symptoms, confirming a statistically significant association between vitamin D and DED [122].

## 6. Vitamin D Supplementation to Manage DED

After oral intake, vitamin D, both in the form of ergocalciferol and cholecalciferol, is rapidly and well absorbed in the small intestine, with plasma levels peaking around 24 h after administration [128]. More sustainable concentrations of serum 25(OH)D seem to be associated with the administration of cholecalciferol with respect to ergocalciferol [129], and, depending on the administered dose, the maximum concentration of 25(OH)D is reached after approximately 7 to 14 days [128]. Not so long ago, simple passive diffusion was thought to be the only mechanism underlying the absorption of vitamin D. However, according to recent in vivo and in vitro studies, vitamin D absorption might also rely on mechanisms mediated by membrane carrier proteins, and, more specifically, cholesterol transporters might be involved [130,131,132]. In addition, it has been observed that the concomitant intake of fat-containing food might improve the absorption of vitamin D [133,134].

Although vitamin D supplementation has been widely proposed for the treatment of DED, the number of clinical studies examining its impact on the disease is still limited. An observational study found that the intramuscular administration of vitamin D had several positive effects in patients who had vitamin D deficiency and were affected by DED that was refractory to artificial tear treatment. Such benefits included: (i) the promotion of tear secretion, (ii) the reduction of tear instability and inflammation at the ocular surface and eyelid margin, and (iii) the improvement of DED symptoms [95]. Improved tear hyperosmolarity, an important sign of DED, was reported in vitamin D-deficient patients after vitamin D systemic replacement therapy [123], and vitamin D oral supplementation was found to improve the health of the ocular surface in patients with vitamin D deficiency [124]. An interesting study tied together low vitamin D levels, dry eye symptoms, and vitamin D supplementation. Although the number of patients was relatively small, the results of this investigation suggested the existence of an association between low vitamin D concentrations and dry eye symptoms. Moreover, the systemic supplementation of vitamin D, by increasing the vitamin D serum levels, was capable of improving ocular surface conditions, tear quality, and more in general, dry eye symptoms [135]. Vitamin D systemic supplementation was also found to enhance the efficacy of topical lipid-containing carbomer-based artificial tears and hyaluronate in patients with DED, suggesting that the effect of topical lubricants may depend on the serum concentrations of vitamin D [125] and to lead to earlier and significant improvements in tear break-up time, Schirmer’s, and OSDI scores in vitamin D-deficient patients with DED [126]. Taken together, these findings seem to suggest that, indeed, patients suffering from DED can benefit from a systemic administration of vitamin D.

## 7. Conclusions

Although it cannot be defined as a systematic review, in our work, we methodically examined the literature providing evidence of the role of vitamin D levels in individuals suffering from DED symptoms. Following such an analysis, it can be concluded that, possibly because of its positive effect in modulating the immune and inflammatory response, the systemic supplementation of vitamin D should be considered as a potentially effective therapeutic strategy, especially, but not only, for patients affected by DED that does not respond to conventional treatment. In this regard, considering the hormonal role of vitamin D and the sigmoidal shape of its dose–response curve, pharmacological doses of vitamin D (>10,000 IU) might be required to restore the severe status of vitamin D deficiency associated with eye diseases. Unluckily, the number of clinical studies examining the vitamin D impact on DED is still limited. Therefore, as future perspectives, appropriate studies should be designed to further investigate this novel therapeutic approach and to deeply investigate the favorable role of vitamin D in maintaining ocular health.

## Figures and Tables

**Table 1:** Clinical studies on the role of vitamin D in DED.

Study	Study Design	Patients	Follow Up	Tools Used	Findings
Kurtul et al., 2015 [114]	Prospective clinical study	34 patients with serum vitamin D deficiency and 21 control subjects	n/a ^1^	The ocular surface disease index (OSDI) questionnaire was used for assessment of dry-eye symptoms. The tear break-up time test (TBUT) and Schirmer test were also evaluated	Vitamin D deficiency decreases the TBUT and Schirmer test values and may be associated with dry-eye symptoms
Demirci et al., 2018 [115]	Prospective clinical study	60 eyes of 30 patients with vitamin D deficiency and 60 eyes of 30 healthy individuals	n/a	Ocular Surface Disease Index (OSDI) questionnaire, Schirmer I test, tear break-up time (TBUT), scoring of ocular surface fluorescein staining using a modified Oxford scale, and tear osmolarity	This study demonstrates that vitamin D deficiency is associated with tear hyperosmolarity and tear film dysfunction and suggests that patients with vitamin D deficiency may be prone to dry eye
Yildirim et al., 2016 [116]	Prospective clinical study	50 premenopausal women with vitamin D deficiency and 48 controls	n/a	Schirmer test, TBUT, OSDI, Stanford Health Assessment Questionnaire (HAQ), fatigue severity scale (FSS), and visual analogue scale-pain (VAS-pain)	Dry eye and impaired tear function in patients with vitamin D deficiency may suggest a protective role of vitamin D in the development of dry eye, probably by enhancing tear film parameters and reducing ocular surface inflammation
Jin et al., 2017 [117]	Retrospective cross-sectional study	A total of 79 patients were included, 22 male and 57 female. The subjects were divided into three groups based on serum 25(OH)D levels: 12 subjects were placed in the sufficient group, 36 subjects in the insufficient group and 31 subjects in the deficient group	n/a	Eye discomfort was measured by the ocular surface disease index (OSDI) and visual analogue pain score (VAS). Tear breakup time (TBUT), and Schirmer tear secretion test were measured to evaluate tear film	This study provides evidence that tear stability and secretion correlate with serum 25(OH)D levels
Meng et al., 2017 [118]	Case–control study	70 DED patients and 70 healthy controls	n/a	Serum 25(OH)D was chosen as the main parameter. DED parameters included ocular surface disease index (OSDI) scales, tear film breakup time (TBUT) and Schirmer test	This study found a significant association between 25(OH)D serum levels and DED incidence
Yoon et al., 2016 [119]	Cross-sectional data analysis	Adults over 19 years of age (N = 17,542) who participated in Korean National Health and Nutrition Examination Survey (KNHANES) 2010–2012	n/a	The components of the KNHANES survey are a health interview, health examination survey, and nutrition survey	Low serum levels of vitamin D and inadequate sunlight exposure are associated with DED in Korean adults
Kim et al., 2017 [120]	Cross-sectional data analysis consolidated from the years 2010 and 2011 of the KNHANES	Adults aged >19 years who participated in KNHANES and underwent ophthalmologic interviews and examinations but didn’t have comorbid conditions associated with dry eye. A total of 9349 participants were included	n/a	The components of the KNHANES survey are a health interview, health examination survey, and nutrition survey	Severe vitamin D deficiency was associated with dry eye in an unadjusted model, but the association was not statistically significant after adjustment
Jeon et al., 2017 [121]	Cross-sectional data analysis	Participants in the Study Group for Environmental Eye Disease (2014–2015). Data from 740 participants (253 men and 487 women) were analyzed	n/a	The association between serum vitamin D levels and DED was evaluated using the ocular surface disease index (OSDI)	The present study provides epidemiological data regarding the absence of an association of serum vitamin D levels and DED in the Korean general population
Liu et al., 2020 [122]	Systematic review and meta-analysis	n/a	n/a	Serum vitamin D levels, OSDI scores, Schirmer’s test and TBUT	This meta-analysis shows that vitamin D deficiency is associated with DED in terms of quantity of tears and vision-related quality of life. Its findings indicate that vitamin D systemic supplementation is a potential therapeutic strategy
Bae et al., 2016 [95]	Observational study	105 patients, 21 men and 84 women, with DED refractory to conventional treatment	2, 6, and 10 weeks after vitamin D supplementation	TBUT, FSS, eyelid margin hyperemia, Schirmer test, OSDI, VAS, and severity and duration of symptoms	Vitamin D supplementation promoted tear secretion, reduced tear instability, and reduced inflammation at the ocular surface and eyelid margin. Vitamin D systemic supplementation also improved the symptoms of DED. In conclusion, vitamin D systemic supplementation is an effective and useful treatment for patients with DED refractory to conventional treatment
Kizilgul et al., 2018 [123]	Prospective clinical study	44 patients, 38 females and 6 males, with vitamin D deficiency	8 weeks after vitamin D replacement	Tear film osmolarity (TFO)	Tear film osmolarity, an important indicator of dry eye disease, decreased after successful vitamin D replacement
Karaca et al., 2020 [124]	Prospective clinical study	40 patients, 34 females, and 6 males, with vitamin D deficiency	8, 12, and 24 weeks after vitamin D replacement	Eyelid margin score, meibomian gland expressibility score, Oxford grading, Schirmer test, tear breakup time, tear osmolarity, and Ocular Surface Disease Index score	Vitamin D replacement appeared to improve ocular surface health in patients with vitamin D deficiency
Hwang et al., 2019 [125]	Retrospective, observational study	116 patients with DED, 82 women and 34 men. All patients were treated with topical carbomer-basedlipid-containing artificial tears (CLAT) and hyaluronate (HU) and supplemented with vitamin D. Patients were divided in vitamin D deficiency (VDD) group (52 patients), and non-VDD group (64 patients) on the basis of vitamin D serum levels	2 weeks after vitamin D supplementation	Ocular Surface Disease Index (OSDI) score, visual analog pain scale score, lid hyperemia, tear breakup time (TBUT), corneal fluorescein staining score, and Schirmer test	Vitamin D supplementation enhanced the efficacy of topical treatment and can be used as potential adjuvant therapy for patients with DED.
Watts et al., 2020 [126]	Prospective interventional randomized study	90 patients with dry eye symptoms and vitamin D deficiency	At days 15, 30, and 90 of treatment	TBUT, Schirmer’s test, and OSDI score	Vitamin D levels play an important role in patients with dry eye and supplementation of vitamin D patients can lead to earlier and significant improvement in dry eye parameters

^1^ n/a: not available.

## Data Availability

Not applicable.
